# Detection of Autoantibodies to Vascular Endothelial Growth Factor Receptor-3 in Bile Duct Ligated Rats and Correlations with a Panel of Traditional Markers of Liver Diseases

**DOI:** 10.1155/2016/6597970

**Published:** 2016-04-24

**Authors:** Florent Duval, Delia Elva Cruz-Vega, Ivonne González-Gamboa, María Teresa González-Garza, Fernando Ponz, Flora Sánchez, Gabriela Alarcón-Galván, Jorge E. Moreno-Cuevas

**Affiliations:** ^1^Catedra de Terapia Celular, Escuela de Medicina, Tecnológico de Monterrey, Avenida Morones Prieto 3000 Pte., 64710 Monterrey, NL, Mexico; ^2^Centro de Biotecnología y Genómica de Plantas (CBGP), UPM-INIA, Campus de Montegancedo, Autovía M40, Km 38, Pozuelo de Alarcón, 28223 Madrid, Spain; ^3^Servicio de Anatomía Patológica y Citopatología, Hospital Universitario “Dr. José Eleuterio González”, Universidad Autónoma de Nuevo León, Madero y Dr. Aguirre Pequeño, 64460 Monterrey, NL, Mexico

## Abstract

There is a need for new noninvasive biomarkers (NIBMs) able to assess cholestasis and fibrosis in chronic cholestatic liver diseases (CCLDs). Tumorigenesis can arise from CCLDs. Therefore, autoantibodies to tumor-associated antigens (TAA) may be early produced in response to abnormal self-antigen expression caused by cholestatic injury. Vascular endothelial growth factor receptor-3 (VEGFR-3) has TAA potential since it is involved in cholangiocytes and lymphatic vessels proliferations during CCLDs. This study aims to detect autoantibodies directed at VEGFR-3 during bile duct ligation- (BDL-) induced cholestatic injury in rat sera and investigate whether they could be associated with traditional markers of liver damage, cholestasis, and fibrosis. An ELISA was performed to detect anti-VEGFR-3 autoantibodies in sera of rats with different degree of liver injury and results were correlated with aminotransferases, total bilirubin, and the relative fibrotic area. Mean absorbances of anti-VEGFR-3 autoantibodies were significantly increased from week one to week five after BDL. The highest correlation was observed with total bilirubin (*R* ^2^ = 0.8450, *P* = 3.04*e* − 12). In conclusion, anti-VEGFR-3 autoantibodies are early produced during BDL-induced cholestatic injury, and they are closely related to cholestasis, suggesting the potential of anti-VEGFR-3 autoantibodies as NIBMs of cholestasis in CCLDs and justifying the need for further investigations in patients with CCLD.

## 1. Introduction

Cholestasis is defined as a decrease in bile flow. It can arise at the hepatocellular level because of impairment of bile secretion by hepatocytes or at cholangiocellular level, generally by obstruction of bile flow through intra- or extrahepatic bile ducts by gall stones or local malignancies [1]. Cholestasis is the pivotal hallmark of the so-called chronic cholestatic liver diseases (CCLDs), but it may also occur in the advanced stage of other chronic liver diseases (CLDs), such as alcoholic liver disease, nonalcoholic fatty liver disease, and chronic hepatitis B and chronic hepatitis C [2]. When left untreated, cholestasis may drive, in the long term, to tumorigenesis of cholangiocytes [3], the epithelial cells that line bile ducts and normally contribute to the modification of bile volume and composition. This evolution to a malignant phenotype of cholangiocytes, similar to cholangiocarcinoma, takes place through a series of functional and structural changes that affect cholangiocytes, starting early after the initial cholestatic insult by activation, proliferation, and secretion of neuroendocrine factors [4]. Chronic cholestatic liver injury is also accompanied by the development of hepatic fibrosis referring to the inappropriate tissue repair* via* excessive connective tissue deposition in the liver [5], which is a common scenario CCLDs share with all CLDs. Fibrosis is dynamical as it can progress to cirrhosis, a condition hardly reversible with significant morbidity and mortality and growing prevalence worldwide [6]. Cholestasis and fibrosis have enormous economic impact on health care expenditures which further increase when cirrhosis and malignant states are reached [7]. Moreover, current screening methods for cholestasis and fibrosis, especially liver biopsy, have significant limitations [8], thus justifying the exploration of new accurate noninvasive biomarkers (NIBMs) able to early assess cholestasis and fibrosis to estimate the prognosis and determine the surveillance strategies in CCLDs.

Autoantibodies against tumor-associated antigens (TAA) represent promising candidates for NIBMs in liver malignancies, such as cholangiocarcinoma [9], and early states of malignancies, like early chronic cholestatic liver injury. In some case, the mere presence of autoantibodies to TAA may precede the clinical diagnosis of liver cancer [10]. This offers a window of opportunity to intervene and prevent or redirect the course of the disease. In addition, contrastingly to polypeptides, antibodies do not undergo proteolysis in serum, and therefore they are highly stable with half time in the bloodstream ranging from 7 to 30 days depending on the subclass of immunoglobulin [11]. In cholangiocarcinoma patients, autoantibodies directed against p53, heat shock protein 70, enolase 1, and ribonuclease/angiogenin inhibitor 1 have already been reported [12, 13]. The mechanism that triggers the autoantibody response against TAA has still not been elucidated but could be consequent to abnormal self-antigen expression by tumor cells, through chemical alteration, mutation, posttranslational modification, misfolding, aberrant cleavage or localization, and overexposure and/or exposure or spillage of new TAA, in conjunction with the development of an inflammatory reaction within the tumor microenvironment [11, 14]. The elicited autoantibodies oriented to these neoepitopes may be involved in tumor surveillance and regulation, a process that involves activation of immunocompetent cells, leading to tumor cell apoptosis [14]. Nowadays, thanks to the progress in the knowledge of CCLDs, in part through the development of animal models like the bile duct ligation (BDL) model of chronic cholestatic liver injury, new autoantibodies to TAA with potential as NIBM can be discovered.

Even though to date only autoantibodies to vascular endothelial growth factor receptor-2 have been reported in a glioblastoma patient [15], all the proteins from the vascular endothelial growth factor (VEGF) family are potential TAA because of their crucial role in tumor growth. Vascular endothelial growth factor receptor-3 (VEGFR-3), a tyrosine kinase receptor for VEGF-C and VEGF-D, has been shown to play a critical role in the pathogenesis of different proliferative events during CLDs, including that of lymphatic vessels [16, 17] and cholangiocytes [4, 18]. In the latter, the upregulation of VEGFR-3 and secretion of VEGF-C ligand have appeared to mediate the adaptive proliferative response of cholangiocyte to BDL-induced early cholestatic liver injury* via* an autocrine mechanism that involves activation of inositol 1,4,5-triphosphate/[Ca2+]i/protein kinase C alpha and phosphorylation of Src/extracellular signal-regulated kinases 1/2 [18]. Similarly, VEGF-D/VEGFR-3 signaling pathway has been suggested to account for the expansion of the lymphatic vessel network that occurs according to the degree of liver fibrosis in CLDs [16, 19]. Based on these facts, the aim of this work was to explore the production of autoantibodies to VEGFR-3 in BDL-induced cholestatic liver injury and investigate whether absorbances associated with the serum levels of autoantibodies to VEGFR-3 are correlated with traditional markers of liver damage, cholestasis, and fibrosis.

## 2. Materials and Methods

### 2.1. Animals

Thirty-six male Wistar rats (300–400 g) were included in this study and divided into seven groups. Five rats were used as a control group without operation. Sixteen underwent BDL. These animals were sacrificed one week later (*n* = 5), three weeks later (*n* = 7), and five weeks later (*n* = 4). Fifteen rats underwent sham operation. These animals (*n* = 5 per group) were sacrificed at same times as BDL groups.

All rats were housed in plastic cages and maintained in an environmentally controlled room (22 ± 2°C, 65 ± 10% humidity) with a 12-hour light/dark cycle; food and water were given* ad libitum*. The Institutional Committee on care and use of Experimental Animals at the Tecnológico de Monterrey approved this study with protocol number 2013-Re-001.

### 2.2. Bile Duct Ligation

 All rats that underwent BDL and sham operations received intraperitoneal injection of ketamine at 50 mg/kg (Anesket, Pisa Agropecuaria, Hidalgo, Mexico) and xylazine at 10 mg/kg (Sedaject, Vedilab, DF, Mexico) to induce anesthesia. In BDL groups, a laparotomy was performed, and the common bile duct was double-ligated with 4-0 silk and cut between the ligatures. In rats that underwent sham operation, common bile duct was isolated but not ligated, neither cut. Then* linea alba* and skin were closed with 4-0 Vicryl and 3-0 Prolene, respectively. All surgeries were performed under aseptic conditions.

Before sacrificing, rats were anesthetized by ether inhalation (J. T. Baker, Center Valley, PA, USA) and blood samples were collected by cardiac puncture. Serum was obtained and stored at −20°C. Then, animals were immediately sacrificed by cervical dislocation, and the liver was removed, sectioned, and fixed in 10% neutral buffered formalin for morphometric quantification of fibrosis.

### 2.3. Serum Biomarkers of Liver Injury and Cholestasis

Serum alanine aminotransferase (ALT), aspartate aminotransferase (AST), and total bilirubin (TB) were determined spectrophotometrically using an automatic biochemical analyzer ILab Aries (Instrumental Laboratory, Holliston, MA, USA).

### 2.4. Morphometric Quantification of Fibrosis

Postfixed liver tissues were embedded in paraffin. Liver slices 4 *μ*m thick were prepared and stained with picrosirius red stain kit (Abcam, Cambridge, MA, USA) according to manufacturer's protocol. Collagen content was assessed by morphometric analysis of picrosirius red-stained liver sections. Thirty pictures per animal were taken at 10x magnification with a microscope (Olympus BX41 TF, Melville, NY, USA) and digital camera (Olympus DP20, Melville, NY, USA). Pictures were processed using imaging software (ImageJ 1.49v, National Institutes of Health, USA). Data were expressed as relative fibrotic area.

### 2.5. Indirect Enzyme-Linked Immunosorbent Assay (ELISA)

An indirect ELISA was performed to detect the presence of VEGFR-3 specific autoantibodies in rat sera from control, sham, and BDL groups. Plates (Nunc MaxiSorp, San Diego, CA, USA) were coated with 1.5 *μ*g of VEGFR-3 peptide (LIPPSO, Girona, Spain) diluted in 50 mM sodium carbonate buffer pH 9.6 and incubated overnight at 4°C. After blocking overnight at 4°C with 3% skimmed milk, plates were incubated 2 h at 30°C with rat sera diluted in PBS with 0.05% Tween 20. Then, horseradish peroxidase-conjugated rabbit anti-rat (Abcam, Cambridge, MA, USA) diluted 1 : 5000 was added and incubated for 45 min at 30°C. Horseradish peroxidase activity was assessed by adding 1-Step ABTS Substrate Solution (Life Technologies, Waltham, MA, USA). Optical density of samples was determined at 405 nm (Tecan Genios Pro, Männedorf, Switzerland). Negative and positive controls were included under the same conditions. Each sample was tested as duplicates.

### 2.6. Statistics Analysis

All data represent mean ± standard deviation. A MANOVA was employed using six dependent variables (liver/body weight ratio, AST, ALT, TB, absorbance relative to the serum level of autoantibodies to VEGFR-3, and relative fibrotic area) with groups corresponding to the control, the three respective sham and BDL procedures with endpoints after one, three, and five weeks. A linear model with fixed effects was adjusted to each dependent variable using the control group as the intercept. Then, comparisons between consecutive BDL groups and between BDL and equivalent sham groups were performed. Furthermore, linear regressions were performed with the absorbance relative to the serum levels of autoantibodies to VEGFR-3 as the response variable and AST, ALT, TB, and the relative fibrotic area as regressors. Finally, Benjamin-Hochberg corrections were employed to control the false discovery rate, which was defined to be at the 0.05 level. All statistical analyzes were performed using R package.

## 3. Results

### 3.1. Validation of the BDL Animal Model

A panel of traditional markers of BDL-induced liver injury (AST and ALT), cholestasis (TB), and fibrosis (relative fibrotic area) was assessed in order to validate the BDL animal model (Table 1). As expected, liver/body weight ratio, AST, ALT, TB, and the relative fibrotic area were all significantly elevated in the three BDL groups when compared to control group and remained significantly higher than equivalent sham groups throughout the experiment. Although a tendency of increment according to the time of BDL is observed for all the studied markers during the five weeks of the experiment, only differences in the relative fibrotic area between all consecutive groups and in the liver/body weight ratio between the 1st week and the 3rd week reached significance. Furthermore, no difference was significant between the control and sham groups for all the studied markers. Therefore, our BDL animal model was validated as an accurate model of liver injury, cholestasis, and fibrosis.

### 3.2. Serum Autoantibodies to VEGFR-3 Are Produced during BDL-Induced Chronic Cholestatic Liver Injury

**Figure 1:**
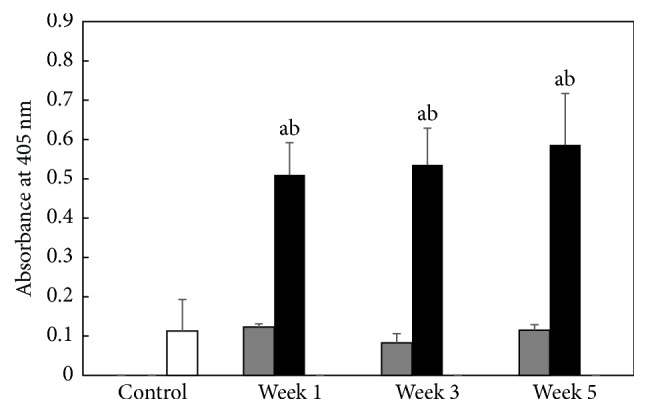
Comparative evaluation of the absorbances relative to the serum levels of autoantibodies to VEGFR-3 by indirect ELISA. Optical density was measured at 405 nm in control group (white bar) (*n* = 3), BDL groups (black bars) at week 1 (*n* = 5), week 3 (*n* = 7), and week 5 (*n* = 4), and sham groups (grey bars) at week 1 (*n* = 3), week 3 (*n* = 3), and week 5 (*n* = 3). ^a^ *P* < 0.05 for BDL and sham groups versus control group; ^b^ *P* < 0.05 for BDL groups versus equivalent sham groups. Data were expressed as mean ± standard deviation. BDL: bile duct ligation.

The analysis of indirect ELISA showed that, in every BDL group, the mean of absorbances associated with the serum levels of autoantibodies to VEGFR-3 is significantly higher than control and equivalent sham groups (Figure 1). At the 1st week, the mean of absorbances increased up to 4 times compared to the control group (mean value of absorbance at the 1st week after BDL: 0.5103 ± 0.0814; *P* = 1.69*e* − 06). The mean of absorbances continued to increase at the 3rd week (mean value of absorbance at the 3rd week: 0.5352 ± 0.0935) and reached a maximum level at the 5th week (mean value of absorbance at the 5th week: 0.5864 ± 0.1307) in BDL groups. Nevertheless, no significant difference between consecutive BDL groups was reached. Means of absorbances associated with the serum levels of autoantibodies to VEGFR-3 of sham groups was not significantly different from that of the control group.

### 3.3. Absorbances Associated with the Serum Levels of Autoantibodies to VEGFR-3 Are Correlated to AST, ALT, TB, and the Relative Fibrotic Area

**Figure 2:**
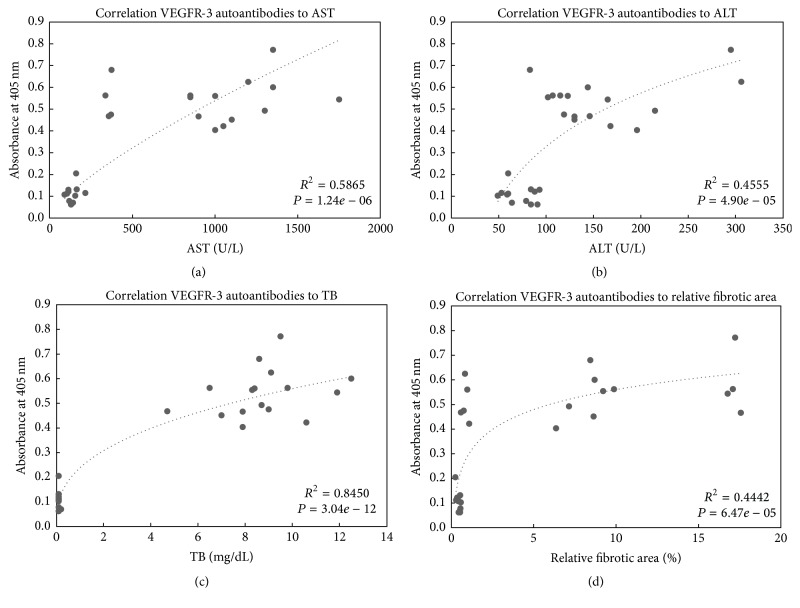
Correlations between absorbances associated with the serum levels of autoantibodies to VEGFR-3 and a panel of traditional markers of liver injury, cholestasis, and fibrosis measured in serum and liver. Correlations between absorbances associated with the serum levels of VEGFR-3 autoantibody obtained in the indirect ELISA and (a) serum AST activity, (b) serum ALT activity, (c) TB serum levels, and (d) relative fibrotic area in liver obtained in all experimental groups. ALT: alanine aminotransferase; AST: aspartate aminotransferase; TB: total bilirubin; VEGFR-3: vascular endothelial growth factor receptor-3.

Correlations between absorbances associated with the serum levels of autoantibodies to VEGFR-3 from the indirect ELISA and AST (Figure 2(a)), ALT (Figure 2(b)), TB (Figure 2(c)), and the relative fibrotic area (Figure 2(d)) were performed. The highest correlation was observed between absorbances associated with the serum levels of autoantibodies to VEGFR-3 and TB (*R* ^2^ = 0.8450, *P* = 3.04*e* − 12). Absorbances associated with the serum levels of autoantibodies to VEGFR-3 also closely correlated with AST (*R* ^2^ = 0.5865, *P* = 1.24*e* − 06), ALT (*R* ^2^ = 0.4555, *P* = 4.90*e* − 05), and the relative fibrotic area (*R* ^2^ = 0.4442, *P* = 6.47*e* − 05).

## 4. Discussion

Autoantibodies are not considered anymore as an exclusive hallmark of autoimmune disorders. In fact, autoantibodies are abundant and ubiquitous in the serum of all humans [20]. Using protein microarrays, Nagele et al., 2013, demonstrated that age, gender, and disease influence autoantibodies serum level in human and proposed that disease-induced perturbations in individual autoantibody profiles present an opportunity to detect accurately and diagnose diseases [20]. In CLDs, autoantibodies have been detected first in autoimmune liver diseases, such as primary biliary cirrhosis and primary sclerosing cholangitis, the clinically most important CCLDs, and more recently in viral hepatitis, drug-induced hepatitis, alcoholic liver disease, nonalcoholic fatty liver disease, hepatocellular carcinoma, and cholangiocarcinoma [21]. Autoantibodies detected in CLDs target a wide repertoire of host antigens, which include proteins from the nucleus, the cytoskeleton, liver kidney microsomes, the mitochondria, and the cytoplasm of neutrophilic granulocytes, as well as liver soluble antigens, asialoglycoprotein receptors, TAA, and stress proteins [21]. However, this list is continuously growing thanks to the advances in the understanding of the pathogenesis of CLDs and the improvement of conventional technologies (ELISA, indirect immunofluorescence, and immunoblotting) for the detection of autoantibodies as well as the development of new efficient technologies (laser bead immunoassays, antigen microarrays, and line immunoassays), which offer the opportunity to manage a higher number of samples with lower costs.

In the present study, we focused on autoantibodies directed against TAA since cholestasis and CCLDs have been associated with liver and biliary tree malignancies, such as cholangiocarcinoma and hepatocellular carcinoma. Hepatocellular carcinoma may arise in the long term of chronic cholestatic liver injury. However, this causal relationship seems unusual as only 1–12% of patients with hepatocellular carcinoma manifest obstructive jaundice as the initial complaint [22]. Cholestasis with chronic inflammation has also been linked to carcinogenesis in cholangiocarcinoma. In combination, both conditions can promote the four major cancer phenotypes: (1) autonomous cell proliferation; (2) invasion/metastases; (3) escape from senescence; and (4) evasion of cell death [23]. Moreover, it has been demonstrated that malignant cholangiocytes proliferate more rapidly after BDL, thereby emphasizing the role of obstructive cholestasis as a potent promotor of intrahepatic cholangiocarcinoma growth and progression [24]. The mechanism of autoantibody induction against TAA is still an area of debate. Autoantibodies are probably induced because cells undergoing malignant transformations express self-antigens that were not evident before [14]. Another explanation is that, during the earliest stage of tumorigenesis, proteins are likely mutated, overexpressed, posttranslationally modified, misfolded, aberrantly cleaved, or aberrantly localized in tumor cells [11]. Under these conditions, altered proteins may pass from an unreactive state to TAA, thus eliciting the production of autoantibodies to try to control the growth of the TAA-expressing tumor.

In particular, we focused on VEGFR-3, the specific receptor for VEGF-C and VEGF-D, because (1) it is already known that VEGF-C/VEGF-D/VEGFR-3 signaling pathway is crucial for the growth and maintenance of tumors, including cholangiocarcinoma [25, 26] and hepatocellular carcinoma [27]; (2) VEGFR-3 is involved in cell proliferation during chronic cholestatic liver injury [18]; (3) VEGFR-3 expression is altered in response to chronic cholestatic liver injury [18, 28]; and (4) structural and functional changes occur in VEGFR-3-expressing cells during chronic cholestatic liver injury [4, 19]. In response to obstructive cholestatic liver injury, cholangiocytes evolve according to three interrelated phenotypes [4]. Early after BDL, an ischemic-reperfusion phenotype, characterized by cholangiocyte depolarization, abnormal ion transport, hypometabolism, and hepatocytic apoptosis, is induced [4]. This phenotype, in turn, could trigger the activation of cholangiocytes which start to proliferate and secrete neuroendrocrine factors to compensate for the loss of biliary cells and sustain secretory activities [3, 4]. In the long term, cholestatic injury drives to tumorigenesis, where the tumorous tissue principally consists of cholangiocyte parenchyma [4]. A study of Gaudio et al. revealed that this adaptive proliferative response of cholangiocyte to cholestasis is mainly mediated by the upregulation of VEGFR-3 and secretion of VEGF-C, through activation of inositol 1,4,5-triphosphate/[Ca2+]i/protein kinase C alpha and phosphorylation of Src/extracellular signal-regulated kinases 1/2, during the first week after BDL injury [18]. The increased proliferation of cholangiocytes in normal rats chronically treated with recombinant VEGF-C and the decreased cholangiocyte proliferation when VEGF is immunoneutralized by antibodies to VEGF-C sustain this theory [18]. Along with cholangiocyte proliferation, VEGF-D/VEGFR-3 signaling pathway has also been implicated in the pathologic reactivation of lymphangiogenesis during CLDs [17]. In patients with viral forms of CLDs, the numbers of lymphatic vessels and their areas are increased according to the degree of fibrosis [19]. Therefore, VEGFR-3 may be converted in TAA during the early steps of the progression of CCLDs towards tumorigenesis. For that reason, we investigated the production of autoantibodies to VEGFR-3 in the BDL-model of chronic cholestatic liver injury.

To determine whether BDL induces a measurable production of VEGFR-3 autoantibodies in serum, we performed an indirect ELISA by coating wells with VEGFR-3 peptide [29]. We found that absorbances associated with the serum levels of autoantibodies to VEGFR-3 in BDL groups were systematically higher than control rats. This result demonstrates that BDL triggers the serum production of autoantibodies to VEGFR-3, which is consistent with the study of Thomas et al., 2012, who reported that the lack of lymphatic drainage, as occurs in the BDL animal model, promotes autoimmunity [30]. Absorbances associated with the serum levels of autoantibodies to VEGFR-3 in BDL groups increased significantly as soon as the 1st week after BDL when compared to control group. This may offer the opportunity of using autoantibodies to VEGFR-3 to early diagnose BDL-induced chronic cholestatic liver injury. Moreover, we showed that these absorbances kept on increasing throughout the five weeks of the experiment even though no significance was reached between consecutive BDL groups, thus suggesting that autoantibodies to VEGFR-3 could be appropriate to monitor the progression of BDL-induced chronic cholestatic liver injury.

Studies on the identification of new NIBMs have revealed that autoantibodies to TAA represent promising candidates [12]. For example, it has been demonstrated that plasma levels of IgG autoantibodies against heat shock protein 70 are accurate to diagnose cholangiocarcinoma and that specificity increases by adding autoantibodies to enolase-1 and ribonuclease/angiogenin inhibitor 1 [12]. Performing correlations between the serum levels of a potential biomarker and traditional markers of CLD pathologic events is a valid strategy for the study of new NIBMs [31]. Therefore, to give the first evidence about the potential of autoantibodies to VEGFR-3 as NIBMs of BDL-induced liver injury, cholestasis, and fibrosis, we performed correlations between absorbances associated with the serum levels of autoantibodies to VEGFR-3, obtained in the ELISA, and a panel of traditional markers of liver injury (AST and ALT), cholestasis (TB), and fibrosis (relative fibrotic area). AST and ALT are NIBMs that reflect hepatic injury, providing valuable information regarding the overall hepatic function [32]. The analysis of aminotransferase serum activity is commonly used as a first step in the evaluation of patients with suspected CLD along with the determination of other serum components. Bilirubin is a marker of cholestasis [33]. A TB test is generally ordered to help diagnose CCLD, such as PSC and PBC. Moreover, hyperbilirubinemia, which characterizes severe CCLDs, has been recognized as a major predictor of clinical outcomes in both PBC and PSC [34, 35]. The relative fibrotic area, obtained by computer-assisted image analysis of picrosirius red-stained liver sections, is representative of the liver deposition of collagen I and collagen III, two of the main components of the extracellular matrix. Progressive accumulation of collagen proteins occurs during liver fibrosis progression [36]. Therefore, the relative fibrotic area can be used to distinguish the different stages of liver fibrosis [37]. In liver diseases, excessive collagen deposition leads to structural disruption of the normal liver architecture, liver stiffness, portal hypertension, and eventually hepatic failure [38–40]. In consequence, methods to supervise the progression of fibrosis are crucial to implement treatment strategies to prevent such events. In this study, we showed that correlations between absorbances relative to the serum levels of autoantibodies to VEGFR-3 and AST, ALT, TB, and the relative fibrotic area were all significant. These results prove that autoantibodies to VEGFR-3 are correlated to BDL-induced liver injury, cholestasis, and fibrosis and support the need to further study these autoantibodies in more types of animal models of liver injury, such as the carbon tetrachloride model, as well as in human subjects with CCLD. Interestingly, the correlation with TB was the highest with *R* ^2^ of 0.845. This finding, in parallel to the observation that the mean value of absorbances associated with the serum levels of autoantibodies to VEGFR-3 is increased directly after BDL and stays high without enabling the distinction between one, three, and five weeks of BDL, a common tendency to TB but not to the relative fibrotic area, suggests that levels of autoantibodies to VEGFR-3 may be better associated with cholestasis than fibrosis. This provides the first evidence on the potential application of autoantibodies to VEGFR-3 as NIBMs of cholestasis in CCLDs. To confirm that, further studies on human patients with CCLD are needed.

## 5. Conclusion

This* in vivo* study demonstrated that BDL promotes the production of VEGFR-3 specific autoantibodies that are early measurable in the serum as soon as the first week after BDL-induced chronic cholestatic liver injury. Although serum levels of autoantibodies to VEGFR-3 significantly correlated with markers of liver damage and fibrosis, the highest correlation was with TB, suggesting that autoantibodies to VEGFR-3 are closely related to BDL-induced cholestasis.

## Figures and Tables

**Table 1:** Measurements of traditional markers of liver injury and cholestasis in serum and fibrosis in the liver.

	Control	Sham			BDL		
	(*n* = 5)	Week 1 (*n* = 5)	Week 3 (*n* = 5)	Week 5 (*n* = 5)	Week 1 (*n* = 5)	Week 3 (*n* = 7)	Week 5 (*n* = 4)
Liver/body weight ratio (%)	3.98 ± 0.32	3.87 ± 0.47^b^	4.07 ± 0.17^b^	3.68 ± 0.32^b^	5.35 ± 0.40^a^	6.99 ± 1.02^ac^	6.84 ± 0.65^a^
AST (U/L)	141.20 ± 13.93	160.20 ± 37.16^b^	117.40 ± 22.07^b^	126.20 ± 23.79^b^	862.83 ± 394.96^a^	974.71 ± 330.69^a^	1084 ± 607.64^a^
ALT (U/L)	71.60 ± 13.05	71.80 ± 14.86^b^	67.60 ± 13.13^b^	72.20 ± 18.30^b^	172.83 ± 69.08^a^	139.57 ± 49.43^a^	176.25 ± 81.89^a^
TB (mg/dL)	0.12 ± 0.05	0.10 ± 00.00^b^	0.10 ± 00.00^b^	0.08 ± 0.05^b^	7.62 ± 2.68^a^	8.97 ± 1.77^a^	8.95 ± 2.32^a^
Relative fibrotic area (%)	0.4292 ± 0.1239	0.45 ± 0.15^b^	0.50 ± 0.05^b^	0.45 ± 0.11^b^	0.84 ± 0.19^a^	8.33 ± 1.20^ac^	17.17 ± 0.33^ac^

Data were expressed as mean ± standard deviation. ^a^ *P* < 0.05 for BDL and sham groups versus control group; ^b^ *P* < 0.05 for BDL groups versus equivalent sham groups; and ^c^ *P* < 0.05 for BDL at week 3 versus BDL at week 1 and BDL at week 5 versus BDL at week 3. ALT: alanine aminotransferase; AST: aspartate aminotransferase; BDL: bile duct ligation; TB: total bilirubin.
